# Overexpression of p54^nrb^/NONO induces differential *EPHA6* splicing and contributes to castration-resistant prostate cancer growth

**DOI:** 10.18632/oncotarget.24063

**Published:** 2018-01-08

**Authors:** Ryuji Yamamoto, Tsuyoshi Osawa, Yusuke Sasaki, Shogo Yamamoto, Motonobu Anai, Kouji Izumi, Yoshihiro Matsumura, Juro Sakai, Hiroyuki Aburatani, Atsushi Mizokami, Tatsuhiko Kodama, Toshiya Tanaka

**Affiliations:** ^1^ Laboratory for Systems Biology and Medicine (LSBM), Research Center for Advanced Science and Technology (RCAST), The University of Tokyo, Tokyo 153-8904, Japan; ^2^ Division of Genome Science, Research Center for Advanced Science and Technology (RCAST), The University of Tokyo, Tokyo 153-8904, Japan; ^3^ Department of Integrative Cancer Therapy and Urology, Division of Cancer Medicine, Graduate School of Medical Science, Kanazawa University, Kanazawa, Ishikawa 920-8641, Japan; ^4^ Division of Metabolic Medicine, Research Center for Advanced Science and Technology (RCAST), The University of Tokyo, Tokyo 153-8904, Japan; ^5^ Division of Molecular Physiology and Metabolism, Tohoku University Graduate School of Medicine, Sendai 980-8574, Japan

**Keywords:** CRPC, neuroendocrine prostate cancer, p54^nrb^/NONO, RNA splicing, EPHA6

## Abstract

The non-POU domain-containing octamer binding protein p54^nrb^/NONO is a multifunctional nuclear protein involved in RNA splicing, processing, and transcriptional regulation of nuclear hormone receptors. Through chromosome copy number analysis via whole-exome sequencing, we revealed amplification of the chromosome Xq11.22-q21.33 locus containing the androgen receptor (*AR*) and *NONO* genes in androgen-independent, castration-resistant prostate cancer (CRPC)-like LNCaP-SF cells. Moreover, *NONO* was frequently amplified and overexpressed in patients with CRPC. RNA sequencing data revealed that a truncated ephrin type-A receptor 6 (*EPHA6*) splice variant (*EPHA6-001*) was overexpressed in LNCaP-SF cells, and knockdown of *NONO* or *EPHA6-001* prevented *EPHA6-001* expression and reduced proliferation and invasion by LNCaP-SF cells grown under androgen deprivation conditions. Growth inhibition and differential splicing of *EPHA6* mRNA by p54^nrb^/NONO were confirmed in gene silencing experiments in 22Rv1 PCa cells. Importantly, *NONO* knockdown in LNCaP-SF cells led to reduced tumor growth in castrated mice. These findings indicate that p54^nrb^/NONO is amplified and overexpressed in CRPC cells and clinical samples, and facilitates CRPC growth by mediating aberrant *EPHA6* splicing. We therefore propose that p54^nrb^/NONO constitutes a novel and attractive therapeutic target for CRPC.

## INTRODUCTION

Prostate cancer (PCa) is one of the most common cancers in men worldwide and a leading cause of cancer mortality [1, 2]. During the early stages of PCa, tumors are usually dependent on androgens for cellular function and growth; accordingly, androgen deprivation therapy (ADT) is the first-line treatment for patients with locally advanced tumors and recurrent or metastatic disease. However, the majority of patients will progress to castration-resistant PCa (CRPC) within 2–3 years after initiation of ADT [3]. Amplification and/or overexpression of the androgen receptor (*AR*) gene is observed in approximately 30% of CRPCs [4, 5], and often sensitizes the AR to its antagonists, causing the latter to exhibit agonistic activity [6]. In addition, several AR mutations and truncated forms, which allow broad-range ligand binding to alternative steroids or mediate constitutive AR signaling, have also been associated with CRPC [5]. Therefore, although the androgen-AR signaling axis still plays a pivotal role in CRPC development, androgen-independent mechanisms also contribute to disease progression [7].

Alternative splicing and associated transcripts play important roles in cancer development, progression, and response to therapy [8–10]. For instance, splicing variants of the AR [5], transient receptor potential melastatin 8 (TRPM8) [11], prolactin receptor (PRLR) [12], epidermal growth factor receptor (EGFR) [13], and vascular endothelial growth factor (VEGF) [14] have been found to regulate cell growth, metastasis, and possibly the epithelial-mesenchymal transition (EMT) in PCa. Specifically, certain constitutively active AR splice variants (AR-Vs), particularly AR-V7, have been implicated in the development of castration resistance [5]. Overexpression of short-form splice variants of TRPM8α, which encode N-terminal fragments of full-length TRPM8, has been shown to enhance migration and invasion in LNCaP cells [11]. Exon-4-deleted EGFR (de4 EGFR) was detected in 27% of PCa samples but not in normal tissue, and its overexpression reduced E-cadherin expression and increased metastatic potential in a glioma cell line [13]. Interestingly, these aberrant truncated variants result from alternative splicing at 3′ splice sites in the most common isoforms, and can confer tumor cells with a more mesenchymal phenotype, resulting in increased potential for migration and invasion. Therefore, cancer-specific splice variants and their regulatory splicing factors are attractive therapeutic targets.

The non-POU domain-containing octamer binding protein p54^nrb^/NONO is a multifunctional nuclear protein involved in RNA splicing, processing, and transcriptional regulation of nuclear hormone receptors. The p54^nrb^/NONO protein belongs to the *Drosophila* behavior human splicing (DBHS) protein family, which includes PTB-associated splicing factor (PSF) and paraspeckle protein component 1 (PSPC1); these proteins perform specific functions by forming homo- or heterodimers. p54^nrb^/NONO possesses conserved N-terminal RNA recognition motifs (RRMs), a NonA/paraspeckle domain (NOPS), and a C-terminal coiled coil [15, 16], and also interacts with various proteins such as transcription factors, RNA polymerase II, splicing factors, exonuclease XRN2, DNA topoisomerase, and Ku70/Ku80 [15–23]. Although p54^nrb^/NONO has been suggested to play a role in cancer development [24, 25], its differential expression and functional involvement in the development and progression of PCa have not been clarified.

We previously showed that human LNCaP-SF cells, which were generated from LNCaP cells under steroid-free conditions, acquire not only androgen-independent characteristics but also an osteoplastic phenotype [26]. In the current study, we aimed to identify the mechanisms underlying the acquisition of androgen-independent cell growth and invasive capacity by using whole-exome sequencing and transcriptome analyses of LNCaP-SF cells and available data from CRPC patients. Our findings provide important insights into the role of p54^nrp^/NONO in RNA splicing and PCa progression to CRPC.

## RESULTS

### Transcriptome analysis of differentially regulated genes affecting CRPC-like properties in LNCaP-SF cells

**Figure 1:**
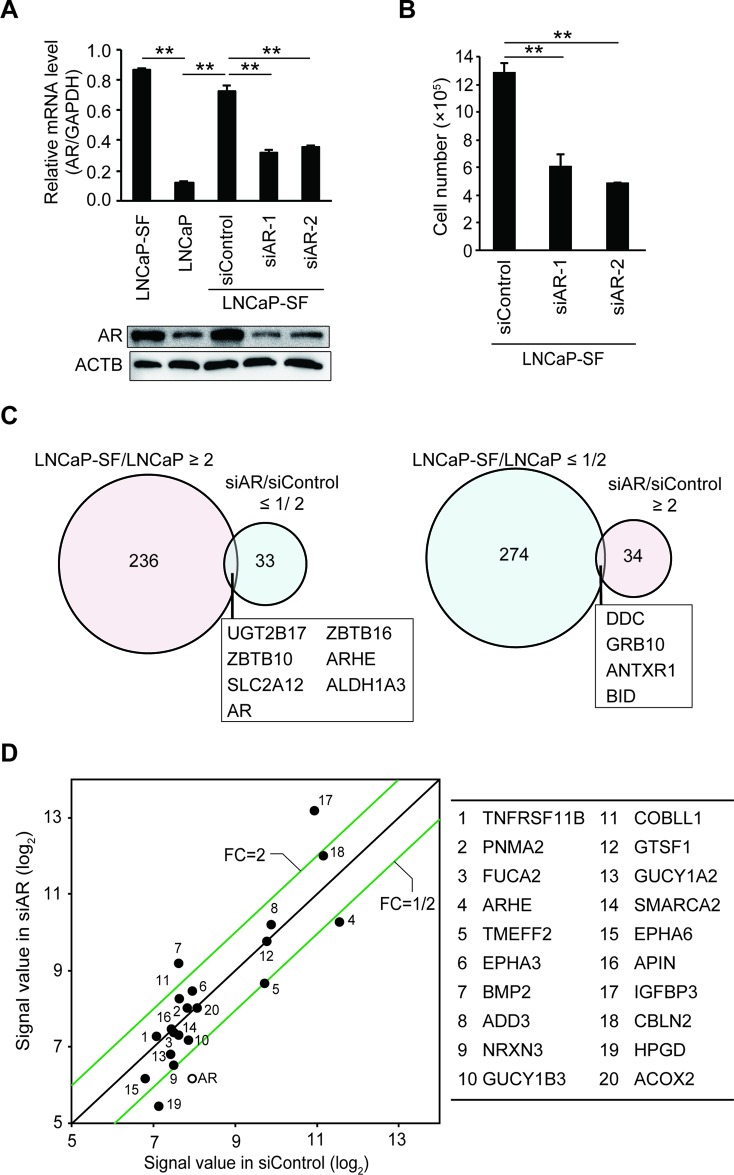
Effect of *AR* silencing on gene expression and growth in LNCaP-SF cells **(A)** Representative qPCR and immunoblot showing that siRNA-mediated AR knockdown effectively reduced AR mRNA and protein expression in LNCaP-SF cells. **(B)** The growth rate of LNCaP-SF cells under androgen deprivation conditions was significantly reduced by siAR transfection. **(C)** Venn diagram showing the overlap of genes differentially expressed in LNCaP-SF/LNCaP cells compared with siAR-regulated transcripts in LNCaP-SF cells. Probe sets labeled light magenta were upregulated, while those labeled light cyan were downregulated. **(D)** Scatter plot analysis of gene expression differences between siRNA control and siAR. The top 20 differentially expressed genes in LNCaP-SF/LNCaP are listed in the right panel; the AR signal value is plotted between the siRNA control and siAR-treated LNCaP-SF cells. FC: fold change. Data represent mean ± s.e.m. ^**^P < 0.01.

LNCaP-SF cells derive from LNCaP cells and were isolated based on their ability to grow in the absence of androgen, thus representing an effective model of CRPC [26]. To clarify the mechanisms underlying the acquisition of androgen-independent cell growth, we first performed comprehensive gene expression profiling on parental (androgen-sensitive) LNCaP cells, and on LNCaP-SF cells transfected with scrambled siRNA (control) or AR-targeting siRNA (AR knockdown) using an oligonucleotide microarray. As reported [27], AR mRNA and protein levels were higher in LNCaP-SF cells compared with parental LNCaP cells (Figure 1A). AR knockdown effectively reduced AR mRNA and protein expression and blocked cell proliferation under androgen-deprivation conditions (Figures 1A-1B), reflecting ligand-binding-domain mutations in the AR gene that confer the corresponding protein with constitutive activity. Compared with parental LNCaP cells, 236 and 274 probe sets were increased and decreased, respectively, in LNCaP-SF cells (Figure 1C and Supplementary Tables 1 and 2). In contrast, 34 probe sets were upregulated, and 33 probe sets were downregulated >2-fold following silencing of AR (Figure 1C and Supplementary Tables 3 and 4). Although the downregulated genes included several known AR targets such as *KLK2*, *KLK3*, *ABHD2*, *ZBTB16*, *HMGCS1*, and *ALDH1A3* [28], we found that only 10 probe sets (7 genes; Figure 1C and Supplementary Table 5) overlapped between upregulated probe sets in LNCaP-SF cells (LNCaP-SF/LNCaP) and downregulated probe sets after transfection with AR siRNA (siAR/siControl LNCaP-SF cells). Similarly, with the exception of the known AR target gene *HPGD*, the top 20 upregulated genes in LNCaP-SF cells were not clearly downregulated by AR silencing (Figure 1D). These upregulated genes included some known to be associated with PCa invasion and bone metastasis, such as *TNFRSF11B*, *EPHA3*, *EPHA6*, and *BMP2* [29–32]. These data indicate the importance of AR-independent signaling in the acquisition of the CRPC-like phenotype in LNCaP-SF cells.

### p54^nrb^/NONO is frequently amplified in CRPC

**Figure 2:**
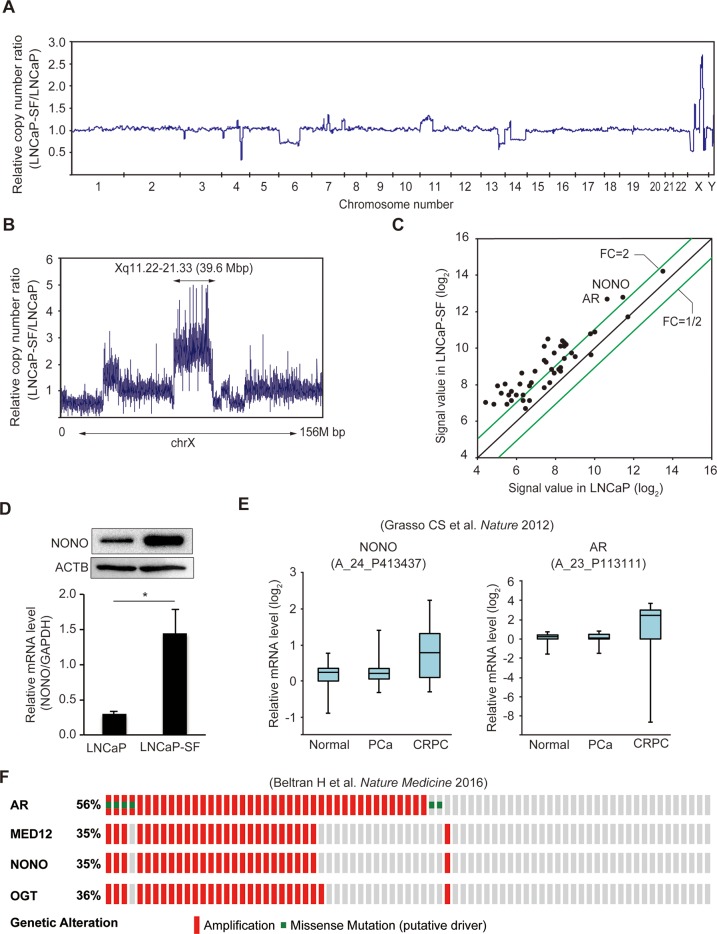
Amplification of the *NONO* gene in LNCaP-SF cells and CRPC samples **(A)** Relative DNA copy number profile of LNCaP-SF cells. **(B)** Relative DNA copy number of chromosome X. Amplification of chromosome Xq11.22-q21.33 was observed in LNCaP-SF cells. **(C)** Scatter plot analysis of genes on chromosome Xq11.22-q21.33 showing expression differences between LNCaP-SF and parental LNCaP cells. **(D)** LNCaP-SF cells show increased p54^nrb^/NONO mRNA and protein expression. **(E)** Relative p54^nrb^/NONO and AR mRNA expression in PCa, CRPC, and normal tissue samples from the Grasso et al. data set (39). p54^nrb^/NONO mRNA expression was upregulated in CRPC. **(F)** Frequency of amplification (red bar) and mutation (green bar) for *AR*, *MED12*, *OGT*, and *NONO* in the Beltran et al. data set (cBioportal; http://www.cbioportal.org) (40). The percentages shown reflect the overall rate of gene amplification and mutation. Vertical aligned bars indicate samples from the same patient. Data represent mean ± s.e.m.

To identify AR-independent signaling molecules associated with CRPC development, genomic differences between parental LNCaP and LNCaP-SF cells were analyzed by whole-exome sequencing. We identified 3,510 somatic mutations (Supplementary Table 6), which included 728 nonsynonymous mutations, 267 synonymous mutations, 110 protein-altering insertions and/or deletions (indels), 4 non-frameshift deletions, 50 gains and/or losses, 1,427 intronic mutations, 129 mutations in the 3′-untranslated region (UTR), 77 mutations in the 5′-UTR, and 718 mutations of other types. Changes in T877A and additional mutations of AR were not observed in LNCaP-SF cells. Chromosome copy number analysis identified 11 regions of copy gains and losses (Figure 2A and Supplementary Table 7) in LNCaP-SF compared with LNCaP cells. In particular, we observed a high level of amplification of the Xq11.22-q21.33 locus (Figure 2B), a region encoding 263 probe sets (Supplementary Table 8), in LNCaP-SF cells. Based on our stringent criteria, 31 of 47 expressed genes were found to be overexpressed in these cells (Figure 2C and Supplementary Table 9), including several ones associated with metastasis progression, such as *AR*, *MED12*, and *OGT* [3–5, 33–35]. In searching for novel genes that might regulate differential gene expression in CRPC, we noted that *NONO*, a gene encoding a multifunctional RNA splicing and processing factor, was located within this amplified region. Indeed, we confirmed by gene-specific qPCR and immunoblotting that p54^nrb^/NONO mRNA and protein were increased in LNCaP-SF cells compared with parental LNCaP cells (Figure 2D). Importantly, these *in vitro* findings were consistent with expression analyses that indicated frequent *NONO* amplification (Supplementary Figure 1) and significant induction of p54^nrb^/NONO mRNA (Figure 2E) in clinical CRPC samples [36]. In addition, amplification of this region was observed in approximately 35% of neuroendocrine PCa specimens (Figure 2F) [37]. Since the neuroendocrine phenotype is closely associated with the development of metastatic CRPC, our findings suggest the importance of *p54^nrb^/NONO* amplification in the acquisition of CRPC-like properties.

### p54^nrb^/NONO regulates the expression of a truncated *EPHA6* splice variant in LNCaP-SF cells

**Figure 3:**
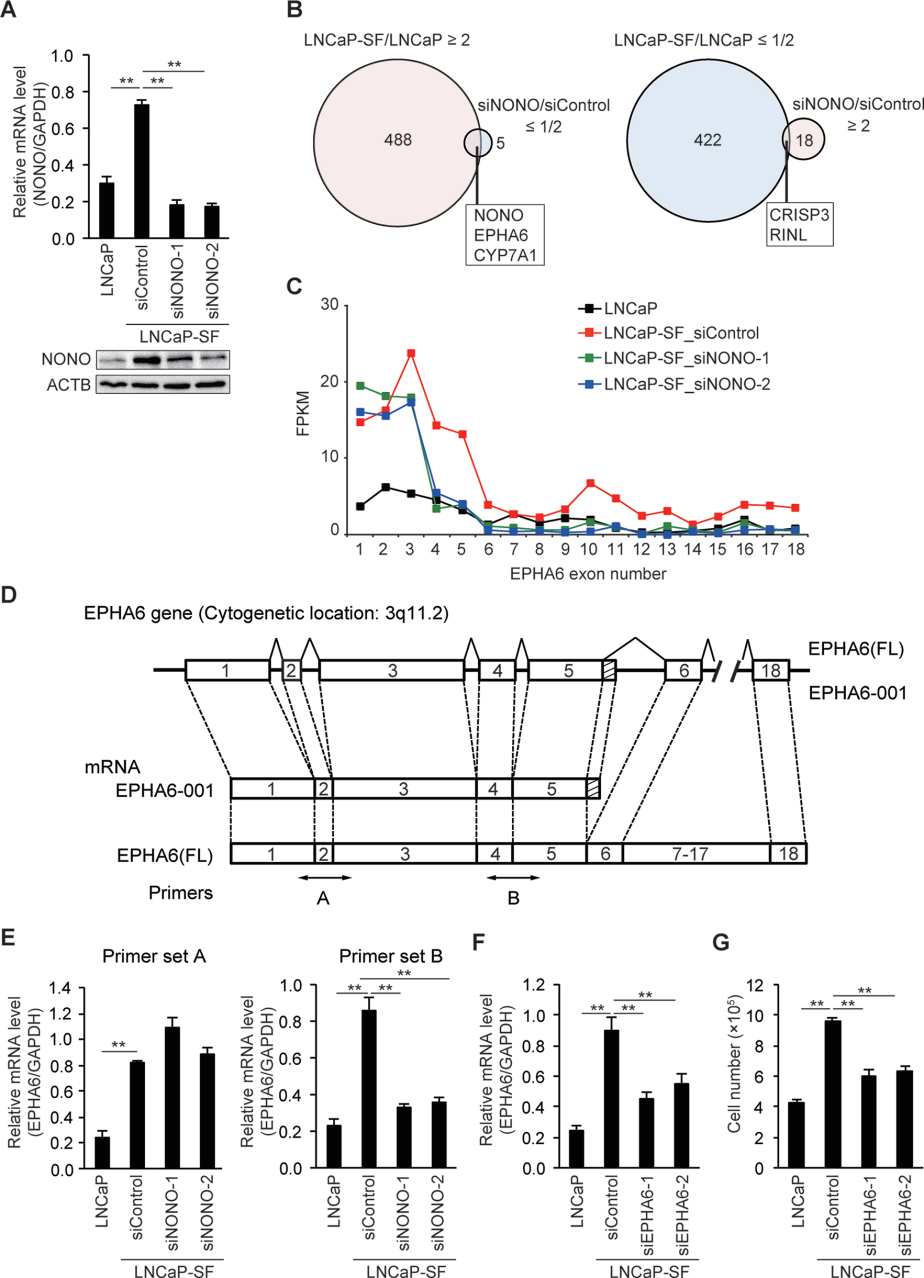
p54^nrb^/NONO mediates differential splicing of the *EPHA6* gene **(A)** Representative qPCR and immunoblot showing that siNONOs effectively reduced p54^nrb^/NONO mRNA and protein expression in LNCaP-SF cells. **(B)** Venn diagram showing the overlap of transcripts differentially expressed in LNCaP-SF/LNCaP cells and siNONO-regulated transcripts. Transcripts labeled light magenta were upregulated, and those labeled light cyan were downregulated. **(C)** Fragments per kilobase of exon per million mapped fragments (FPKM) values of *EPHA6* exons. Exons 1–5 were significantly increased in LNCaP-SF cells. p54^nrb^/NONO silencing specifically reduced the FPKM value corresponding to exons 4 and 5. **(D)** Schematic gene structure comparison between full length *EPHA6* (FL) and its truncated splice variant *EPHA6-001*. Transcription of *EPHA6-001* is initiated from the same first exon of the *EPHA6* (FL) gene and the unique C-terminal sequence of 12 amino acids is encoded by an extended exon downstream of exon 5. Specific primer pairs across exons 1 to 3 (primer set A) and exons 4 and 5 (primer set B) are indicated. **(E)** Effect of siNONOs on *EPHA6-001* expression in LNCaP-SF cells. siNONOs markedly reduced exons 4-5 of mRNA *EPHA6* expression in LNCaP-SF cells (data obtained using the primer set B). **(F)** Effect of *EPHA6* common (siEPHA6-1) and *EPHA6-001* specific (siEPHA6-2) siRNAs on *EPHA6-001* expression in LNCaP-SF cells. **(G)** Transfection with siEPHA6s significantly reduced the growth rate of LNCaP-SF cells under androgen deprivation conditions. Data represent mean ± s.e.m. ^**^P < 0.01.

To evaluate the effects of p54^nrb^/NONO on gene expression, we knocked down p54^nrb^/NONO in LNCaP-SF cells and performed RNA-seq. Treatment with two independent siRNAs resulted in greater than 70% knockdown of p54^nrb^/NONO mRNA and protein in LNCaP-SF cells (Figure 3A). Compared with parental LNCaP cells, 488 and 422 transcripts were upregulated and downregulated, respectively, in LNCaP-SF cells (Figure 3B and Supplementary Tables 10 and 11). In contrast, 18 transcripts were found to be upregulated, and 5 transcripts were found to be downregulated greater than 2-fold after p54^nrb^/NONO silencing (Figure 3B and Supplementary Tables 12 and 13). We found 5 overlapping genes (*NONO, EPHA6, CYP7A1, CRISP3*, and *RINL*) when comparing changes between differentially expressed genes after p54^nrb^/NONO silencing in LNCaP-SF cells and gene expression data from control LNCaP-SF and LNCaP cells (Figure 3B). RNA-seq analyses suggested that p54^nrb^/NONO had only a minor effect on the total number of altered transcripts in LNCaP-SF cells. Instead, we found that silencing of p54^nrb^/NONO induced aberrant RNA splicing of *EPHA6*, which encodes an ephrin receptor reported to be overexpressed and to promote angiogenesis and metastasis in human PCa [31] (Figure 3C). The full-length (FL) *EPHA6* gene consists of 18 exons (encoding 1130 amino acids), while its C-terminally truncated *EPHA6-001* variant (encoding 547 amino acids) is produced by addition of 36 bp (12 amino acids) after exon 5 and a 3′-UTR of 371 bp (Figure 3D). Importantly, RNA-seq results showed that *EPHA6-001* was significantly upregulated in LNCaP-SF cells compared with LNCaP cells, and p54^nrb^/NONO knockdown specifically reduced RNA fragment reads corresponding to exons 4 and 5. To confirm this finding, qPCR was performed using primer sets designed to span an exon-exon junction of *EPHA6* mRNA. As expected, we observed a significant decrease in the expression of transcripts spanning exons 4 to 5 in p54^nrb^/NONO-silenced LNCaP-SF cells (Figure 3E). These results suggest that p54^nrb^/NONO induces aberrant RNA splicing of *EPHA6* to generate the truncated *EPHA6-001* variant, and that reduced *EPHA6-001* expression upon p54^nrb^/NONO silencing is not due to suppressed EPHA6 transcription.

Next, we evaluated the effect of siRNA-mediated silencing of *EPHA6-001* (siEPHA6-2) on the androgen-independent growth of LNCaP-SF cells. Although knockdown efficiency was approximately 35%, *EPHA6-001* silencing in LNCaP-SF cells significantly reduced androgen-independent cell growth by 38% (Figures 3F-3G).

### Knockdown of p54^nrb^/NONO inhibits growth and invasion in LNCaP-SF cells

**Figure 4:**
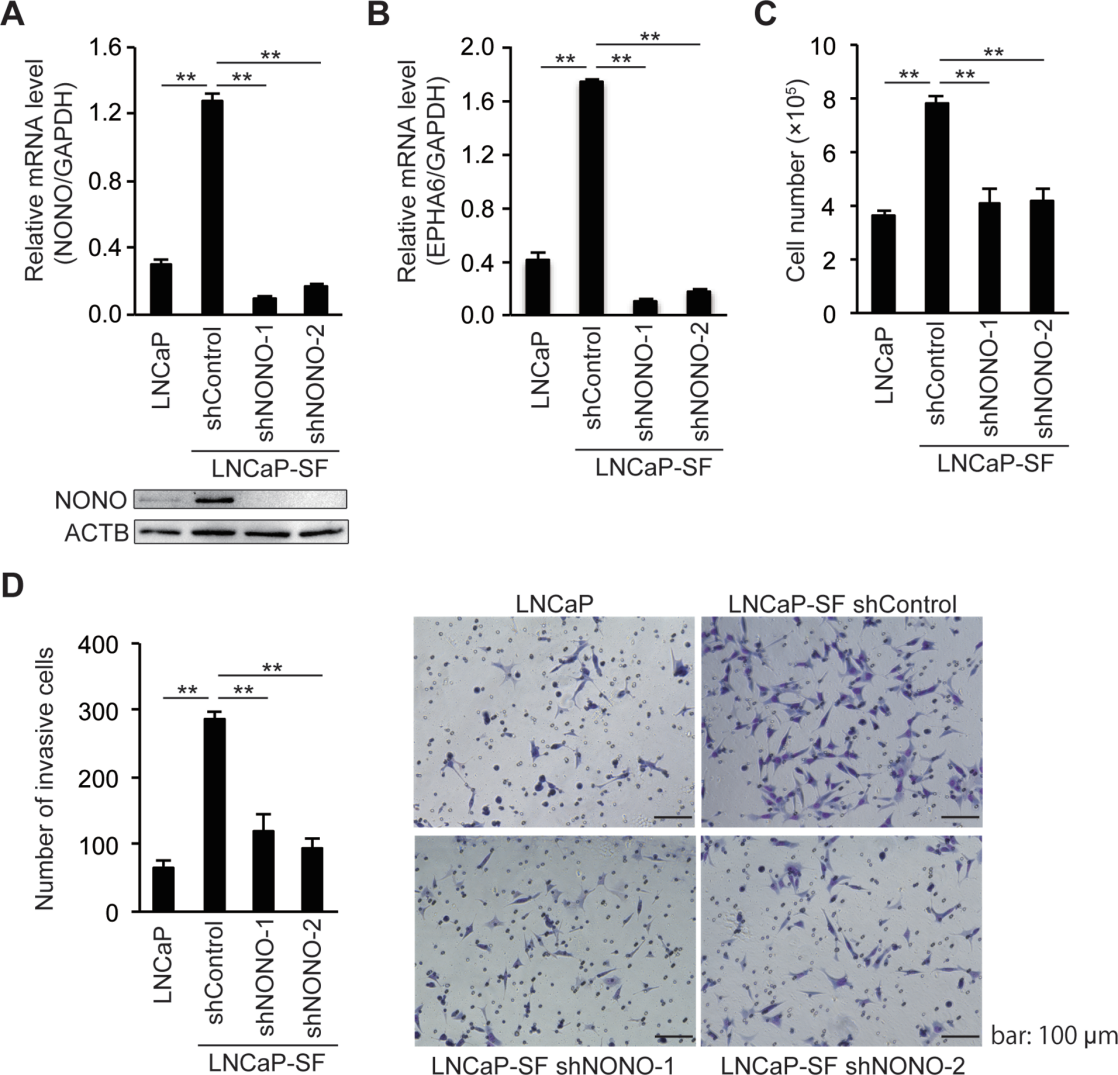
*NONO* silencing reduces LNCaP-SF cell growth and invasion qPCR and immunoblot showing that levels of p54^nrb^/NONO **(A)** and EPHA6 **(B)** transcripts were efficiently reduced in LNCaP-SF cells after transduction with a lentivirus carrying two different shNONOs. **(C)** Transduction with shNONOs effectively reduced the growth rate of LNCaP-SF cells under androgen deprivation conditions. **(D)** Transduction with shNONOs significantly reduced the invasion ability of LNCaP-SF cells. A representative result of the cell invasion assay among the four groups of cells is shown. Data represent mean ± s.e.m. ^**^P < 0.01.

Because tumor growth under androgen deprivation conditions is a hallmark of CRPC, we next assessed the effects of p54^nrb^/NONO on the growth of LNCaP-SF cells under androgen deprivation conditions. We used two independent shRNAs to stably knockdown p54^nrb^/NONO in LNCaP-SF cells. Consistent with the results of siRNA experiments (Figure 3), p54^nrb^/NONO shRNAs effectively suppressed p54^nrb^/NONO mRNA and protein expression and downregulated the p54^nrb^/NONO target *EPHA6-001*, compared with LNCaP-SF cells transfected with untargeted control shRNA (Figures 4A-4B). Compared with parental LNCaP cells, under androgen deprivation conditions LNCaP-SF cells exhibited an accelerated growth rate, which was decreased by p54^nrb^/NONO knockdown (Figure 4C). This result was further supported by the observation that LNCaP-SF cell growth was also suppressed by siRNA-mediated knockdown of p54^nrb^/NONO (Supplementary Figure 2).

Next, to examine the role of p54^nrb^/NONO in the metastatic process, we performed *in vitro* invasion assays by counting the cells that migrated through a Matrigel basement membrane. The number of LNCaP-SF invading cells, which was significantly larger than that of parental LNCaP cells, was reduced after transfection with two independent p54^nrb^/NONO shRNAs (Figure 4D).

**Figure 5:**
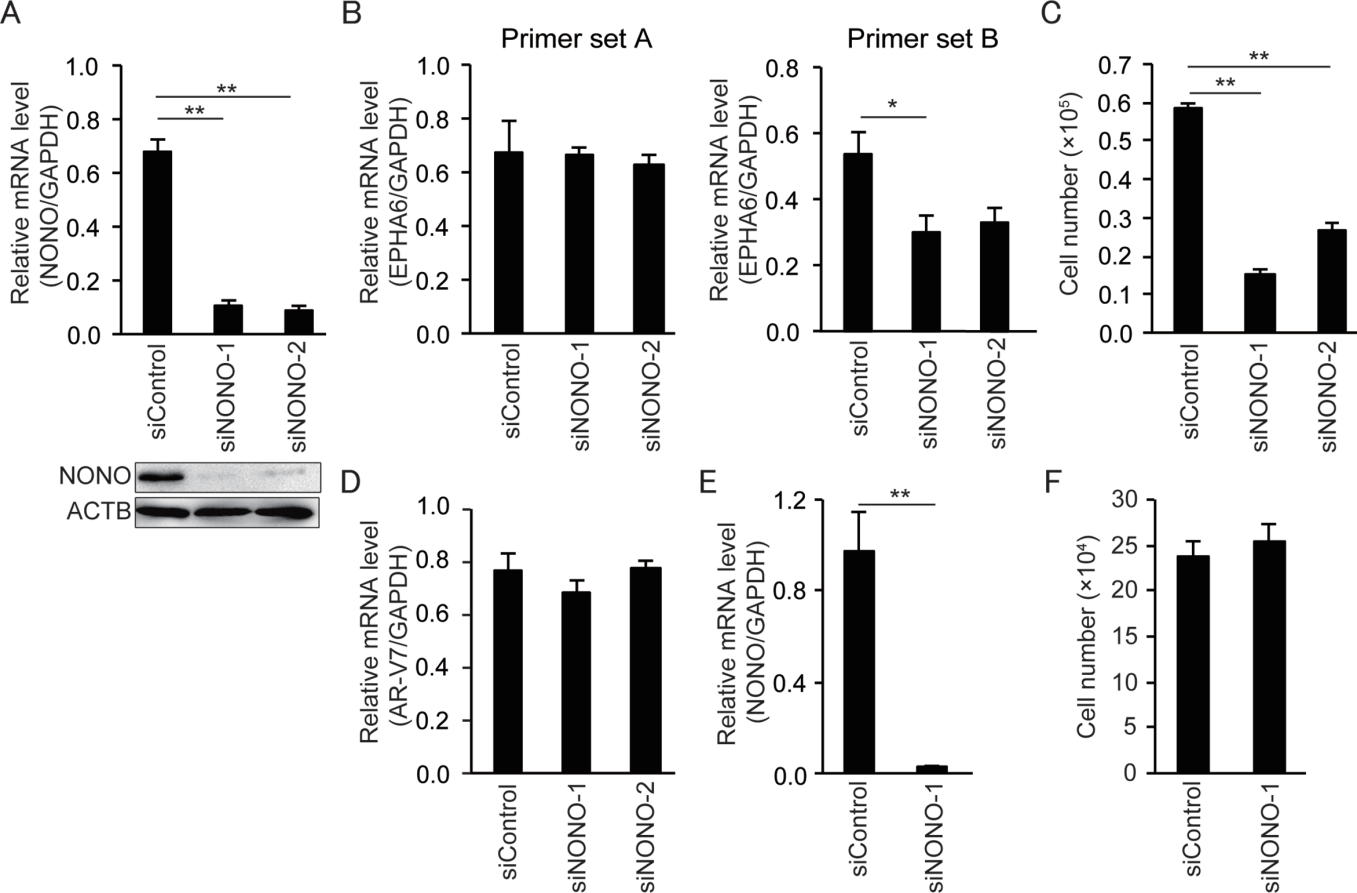
*NONO* knockdown inhibits growth of 22Rv1 cells but not of HUVECs **(A)** Representative qPCR and immunoblot showing that siNONOs effectively reduced p54^nrb^/NONO mRNA and protein expression in 22Rv1 cells. **(B)** Transfection with siNONOs markedly reduced transcripts spanning exons 4 to 5 of *EPHA6* in 22Rv1 cells (data obtained using *EPHA6* primer set B). **(C)** Transfection with siNONOs significantly reduced the growth rate of 22Rv1 cells. **(D)** *AR-V7* expression was not affected by siNONOs in 22Rv1 cells. **(E)** Reduced NONO mRNA expression in siNONO-1-transfected HUVECs. **(F)** No significant reduction of cell growth rate was observed in siNONO-1-transfected HUVECs. Data represent mean ± s.e.m. ^**^P < 0.01.

To further verify the effect of p54^nrb^/NONO on *EPHA6* mRNA splicing and cell growth in CRPC cells, we performed siRNA-mediated p54^nrb^/NONO knockdown in another androgen-insensitive PCa cell line, i.e. 22Rv1 cells (Figure 5A). Similar to LNCaP-SF cells, p54^nrb^/NONO knockdown in 22Rv1 cells induced aberrant *EPHA6* mRNA splicing (Figure 5B) and reduced cell growth rate (Figure 5C), whereas *AR-V7* expression was not changed (Figure 5D). To assess whether similar effects could be observed in non-CRPC cells, p54^nrb^/NONO knockdown was performed in human umbilical vein endothelial cells (HUVECs). Although a significant downregulation of p54^nrb^/NONO mRNA (Figure 5E) and protein expression was achieved, no statistically significant reduction was noted in HUVECs’ growth rate (Figure 5F). On the other hand, *EPHA6* mRNA was essentially undetectable in HUVECs (Ct values above 35). Altogether, these data suggest that the p54^nrb^/NONO-EPHA6 axis may be a specific regulator of proliferation and invasion in CRPC cells.

### p54^nrb^/NONO knockdown suppresses xenograft tumor growth

**Figure 6:**
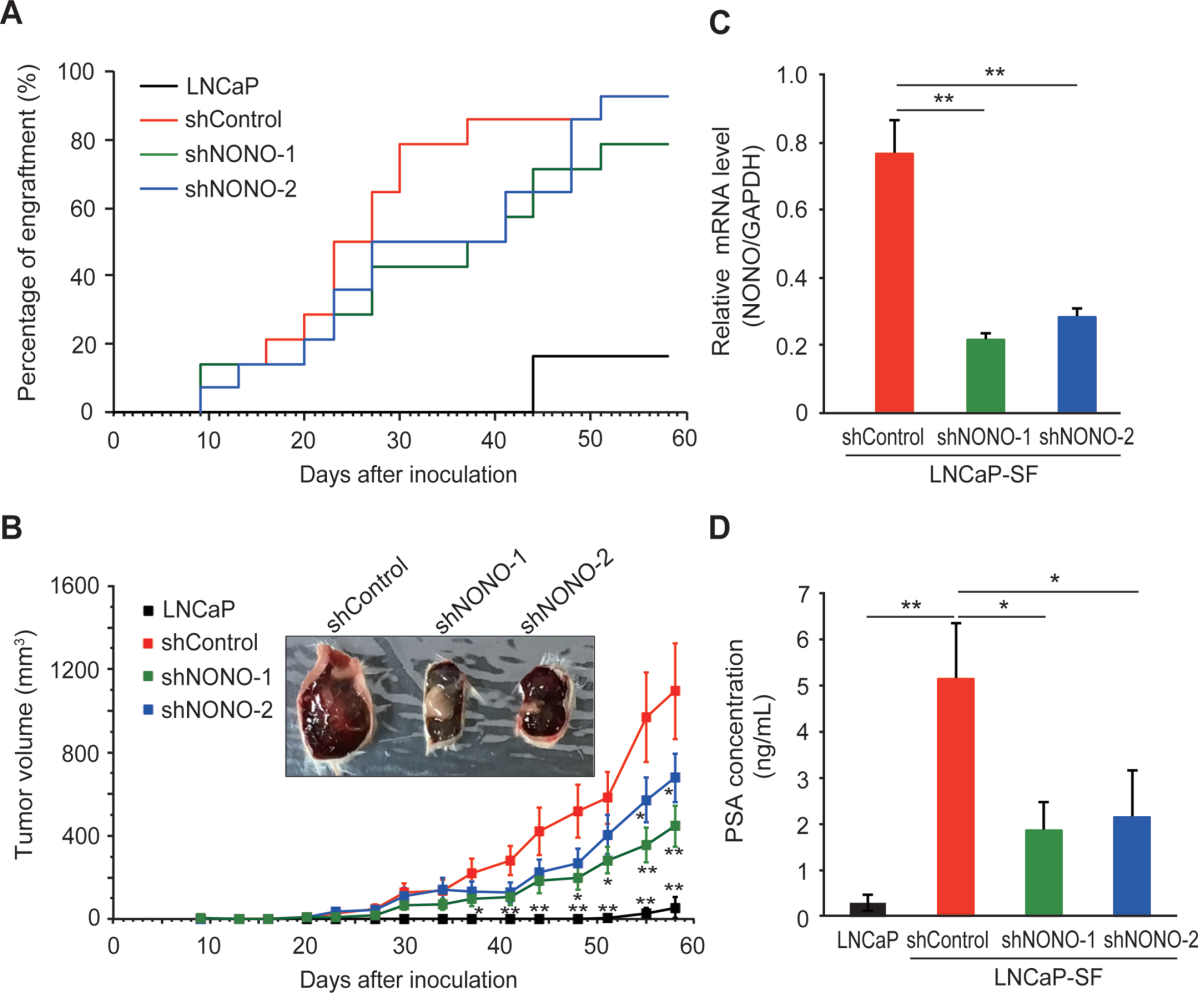
Effect of *NONO* silencing on LNCaP-SF tumor xenograft growth **(A)** Effect of p54^nrb^/NONO shRNAs on engraftment ability of LNCaP-SF cells. Tumor engraftment was scored as positive when tumors were visually detected. **(B)** Effect of p54^nrb^/NONO shRNAs on LNCaP-SF tumor growth. **(C)** p54^nrb^/NONO shRNAs effectively downregulated *NONO* transcription in LNCaP-SF tumors. **(D)** Serum PSA levels were significantly reduced by shNONOs. Data represent mean ± s.e.m. ^*^P < 0.05; ^**^P < 0.01.

We then evaluated the effects of p54^nrb^/NONO knockdown on tumor growth in castrated male mice, in which the residual androgen level is insufficient to maintain the growth of androgen-dependent PCa cells. Castrated male SCID mice were injected subcutaneously with LNCaP-SF cells transfected with shControl, shNONO-1, or shNONO-2 shRNAs and examined every 3 or 4 days for evidence of tumor engraftment and tumor volume measurements. Parental LNCaP cells did not form tumors until 6 weeks after injection, and these tumors grew slowly (Figure 6A). In contrast, LNCaP-SF shControl cells exhibited greater than 90% engraftment efficiency during the experimental period and a rapid growth rate (Figures 6A-6B). Also, tumors formed from LNCaP-SF shControl cells were larger and produced higher levels of serum PSA (Figure 6D) than those derived from parental LNCaP cells. In contrast, p54^nrb^/NONO knockdown in LNCaP-SF cells suppressed tumor formation and reduced growth rate and serum PSA levels (Figures 6A–6D). Taken together, these data suggest that amplification of p54^nrb^/NONO has an important role in the progression of PCa to CRPC.

## DISCUSSION

Since aberrant AR reactivation is commonly involved in the progression of PCa to CRPC, understanding the AR signaling-independent and cooperative mechanisms underlying the transition to CRPC is essential to develop successful therapies. Although many groups have generated and tested androgen-independent PCa cell lines, their findings often lacked clinically relevant, confirmatory data. In the current study, several notable findings were made using the androgen-independent LNCaP-SF cell line established by us [26]. First, amplification of the Xq11.22-q21.33 chromosomal region, which encodes the *AR* gene, was detected in LNCaP-SF cells and verified in CRPC and neuroendocrine PCa clinical samples. Second, Xq11.22-q21.33 amplification correlated with overexpression of p54^nrb^/NONO, which in turn induced overexpression of the truncated *EPHA6* gene variant *EPHA6-001*. Third, p54^nrb^/NONO and *EPHA6* silencing experiments indicated that the p54^nrb^/NONO-*EPHA6-001* axis stimulates the growth of LNCaP-SF cells under androgen deprivation conditions.

X-chromosome genes are known to be frequently associated with specific cancers [38]. A seminal study by Visakorpi et al. reported that 30% of ADT patients with recurrent PCa tumors showed amplification of the Xq11-q13 chromosomal region containing the AR gene [4]. Later, evidence showed that the Xq11.22-q21.33 locus includes not only *AR*, but also *MED12*, *OGT*, and *NONO* [3–5, 33–35]. Although numerous reports indicated that AR amplification drives CRPC [4, 5], other genes located in this region have also been implicated in PCa development and progression. Somatic mutations and overexpression of MED12 have been reported in CRPC [33, 34]. MED12 is a subunit of the Mediator complex, an evolutionarily conserved transcriptional regulator that regulates Wnt, sonic hedgehog (SHH), and transforming growth factor-β (TGFβ) signaling pathways [33, 39, 40]. Similarly, the OGT enzyme, which mediates O-glcNAcylation of multiple proteins, has been shown to be overexpressed in PCa and to play a role in PCa invasion, angiogenesis, and metastasis [35]. Indeed, amplification of *AR*, *MED12*, *OGT*, and *NONO* has been observed in both metastatic and neuroendocrine PCa [36, 37]. Thus, our results suggest that the acquisition of a CRPC-like phenotype in LNCaP-SF cells via Xq11.22-q21.33 amplification resembles the pathogenic events leading to CRPC, and indicate that LNCaP-SF cells are a suitable research tool for studying the mechanisms of CRPC progression and for drug screening.

p54^nrb^/NONO is a multifunctional nuclear protein involved in many aspects of gene expression and RNA splicing and processing [15, 16]. We found, however, that a small number of genes were differentially regulated by p54^nrb^/NONO silencing in LNCaP-SF cells. Homo- and heterodimers of the Drosophila behavior/human splicing (DBHS) protein family members SFPQ, PSPC1, and p54^nrb^/NONO have been shown to interact with transcription factors, DNA, and RNA, to exert various functions. SFPQ/NONO has been reported to bind to nuclear receptors such as thyroid hormone receptor [17], progesterone receptor [41], and steroidogenic factor 1 (SF-1) to negatively regulate their transcription [18]. Also, Yadav et al. reported that NONO is associated with the promoters of many transcriptionally active genes and activates *rhodopsin* and a subset of phototransduction genes [42]. In addition, SFPQ/NONO is required for efficient transcriptional termination, pre-mRNA 3′-end processing, paraspeckle formation, and DNA repair [15, 16]. Li et al. reported that NONO knockout can be rescued by upregulation of the PSPC1 paralog and formation of a functional heterodimer with SFPQ during DNA repair [43]. In accordance with this observation, we found that PSPC1 expression was significantly induced by p54^nrb^/NONO silencing in LNCaP-SF cells. Therefore, PSPC1 might compensate for the loss of p54^nrb^/NONO-regulated gene expression and function. However, our results suggested that other DHBS family proteins may not compensate for specific functions of p54^nrb^/NONO in LNCaP-SF cells including, critically, production of the *EPHA6-001* transcript by alternative RNA splicing. This fact correlates well with evidence that knockdown of p54^nrb^/NONO in the retina specifically induces splicing defects and altered expression of the *rhodopsin* gene [42], and the observation that spontaneous upregulation of PSPC1 and SFPQ could not rescue the behavioral deficits caused by NONO ablation in mice nor the intellectual disabilities caused by NONO mutations in humans [44].

Alternative splicing is a crucial gene regulation mechanism that increases functional proteomic diversity and controls multiple aspects of development. Its dysregulation is implicated in various human diseases including cancer [8–10], where specific splicing isoforms contribute to tumor progression and the acquisition of a metastatic phenotype [11–14]. Along these lines, the present work revealed that a truncated splice variant of the *EPHA6* gene (*EPHA6-001*), is overexpressed in androgen-independent LNCaP-SF cells and contributes critically to proliferation under androgen deprivation conditions. Eph receptors and their ligands, ephrins, regulate important cell communication systems and have widespread roles in both normal physiology and disease, affecting also the growth, migration, and invasion of cancer cells [45]. Recently, Li et al. reported that EPHA6 is consistently overexpressed in metastatic PCa. In their report, shRNA targeting a common region of both full-length and truncated splice variants of *EPHA6* seemed to decrease tumor angiogenesis and metastasis to a larger degree, compared with an shRNA that targeted only full length *EPHA6* [31]. This is consistent with our present data, and suggests that alternative splicing of the *EPHA6* gene plays a critical role in tumor growth and metastasis in CRPC. Similar to *NONO*, *EPHA6* ablation or mutation is associated with behavioral deficits in mice and intellectual disability in humans [46, 47]. Therefore, although the precise mechanisms underlying p54^nrb^/NONO-mediated regulation of *EPHA6* splicing are not fully understood, these observations indicate that the p54^nrb^/NONO-EPHA6 axis has pivotal roles in both neurodevelopment and CRPC progression.

In summary, the current study and previous observations indicate that p54^nrb^/NONO is overexpressed in both AR-independent PCa cells and CRPC samples, and by differentially regulating *EPHA6* splicing and expression it facilitates CRPC growth. Because other DBHS family members may compensate for most of p54^nrb^/NONO cellular functions after its loss, but don't appear to affect *EPHA6* splicing nor expression, p54^nrb^/NONO may be an ideal therapeutic target for CRPC.

## MATERIALS AND METHODS

### Cell culture

The human prostate cancer LNCaP cell line was purchased from the American Type Culture Collection (ATCC) and maintained in Dulbecco's-modified Eagle's medium (DMEM) (Sigma-Aldrich) supplemented with 10% heat-inactivated fetal bovine serum (FBS) containing antibiotics (penicillin, 50 IU/mL; streptomycin, 50 μg/mL) at 37°C in 5% CO_2_. LNCaP-SF cells were established after long-term subculture of parental LNCaP cells in DMEM and 5% charcoal-stripped fetal calf serum (CCS) [26] and maintained in DMEM supplemented with 5% CCS and antibiotics at 37°C in 5% CO_2_. The human prostate cancer 22Rv1 cell line was purchased from ATCC and maintained in Roswell Park Memorial Institute 1640 medium (RPMI 1640; Gibco) supplemented with 10% heat-inactivated FBS containing antibiotics (penicillin, 50 IU/mL; streptomycin, 50 μg/mL) at 37°C and 5% CO_2_. Human umbilical vein endothelial cells (HUVECs) were purchased from KURABO (Cat#: KE-4109P10, Strain No. 04609, Osaka, Japan) and maintained in EGM-2 bullet kit medium (CC-3162, Lonza, Walkersville, MD).

### Small interfering RNA (siRNA) transfection

Silencer^®^ Select siRNAs (Thermo Fisher Scientific) targeting *AR*, *NONO*, *EPHA6*, and *EPHA6-001* were used to inhibit the expression of the corresponding transcripts. The day before transfection, 1 × 10^5^ cells/well were plated in 6-well plates. Cells (LNCaP-SF, 22Rv1, or HUVECs) were transfected with siRNA using Lipofectamine RNAiMAX transfection reagent (Thermo Fisher Scientific) in Opti-MEM (Thermo Fisher Scientific) according to the manufacturer's protocol. All the siRNA sequences used in this study are listed in Supplementary Table 14.

### Short hairpin RNA (shRNA) transduction

To deplete cellular p54^nrb^/NONO, MISSION^®^ Lentiviral Packaging Mix and lentiviral shRNA transfer vectors (Sigma-Aldrich) were cotransfected into 293FT cells using Lipofectamine 2000 (Life Technologies). Briefly, 30 μl of Lentiviral Packaging mix (Sigma-Aldrich), 3 μg of pLKO.1-puro Non-Mammalian shRNA Control, NONO-1 (TRCN0000286628), or NONO-2 (TRCN0000286693), and 1.5 ml of Opti-MEM were mixed. This was combined with a pre-incubated (5 min) mix of Lipofectamine 2000 (36 μl) and Opti-MEM solution (1.5 ml), and incubated for 20 min at room temperature. The DNA-Lipofectamine 2000 complex was added to a 10 cm^2^ plate containing 5 ml of Opti-MEM containing 10% FBS. Five ml of a 1.2 × 10^6^ cells/ml 293FT cell suspension in Opti-MEM containing 10% FBS were added to the plate. Viral supernatants were harvested 72 h after transfection. LNCaP-SF cells were infected with the lentivirus stock with 6 μg/ml of polybrene. Twenty-four hours after transduction, media was replaced with fresh complete media containing 4 μg/ml puromycin to select stable cell clones. All the shRNA sequences used in this study are listed in Supplementary Table 15.

### Exome capture, library construction, and sequencing

Total DNA was extracted from LNCaP-SF cells and parental LNCaP cells using the PureLink™ Genomic DNA Mini Kit (Life Technologies). One microgram of DNA per sample was sheared with a Covaris SS Ultrasonicator. We used a Sciclone NGS workstation (Caliper Life Sciences) for automated library construction. Exome capture was performed with Agilent SureSelect Human All Exon Kit v4 (Agilent Technologies). Each sample was sequenced on an Illumina HiSeq 2000 system using a read length of 2 × 100 bp. Image analysis and base calling were performed using the Illumina pipeline with default settings [48]. Summary statistics and data quality metrics for whole exome sequencing are shown in Supplementary Table 16.

### Exome sequence processing

Exome reads were mapped to the human genome (GRCh37/hg19) using Burrows-Wheeler Aligner (BWA) and Novoalign software independently. Reads with a minimal editing distance to the reference genome were taken to represent optimal alignments. Then, bam files were locally realigned with SRMA. Normal-tumor pair bam files were processed using an in-house genotyper (karkinos; https://sourceforge.net/projects/karkinos/).

### Transcriptome microarray analysis

For genome-wide transcription analysis, the GeneChip Human Genome U133 Plus 2.0 array was used as previously described [49, 50]. Briefly, total RNA was extracted with ISOGEN (Nippon Gene) from LNCaP, LNCaP-SF, and LNCaP-SF cells treated with siRNAs (non-targeting control, siAR-1, and siAR-2). Following *in vitro* transcription (IVT) and cRNA fragmentation, the fragmented IVT product was hybridized on an array and stained with streptavidin phycoerythrin according to the manufacturer's recommended protocol. The arrays were scanned using the Affymetrix GeneChip Scanner 3000 (Affymetrix), and GeneChip Analysis Suite software program version 5.0 (Affymetrix) was used to calculate the signal value for each gene probe.

### Quantitative microarray analysis

Gene expression levels and fold changes between samples were calculated using the GeneChip Analysis Suite software program version 5.0 [49, 50]. The signal value in each experiment was normalized to 100. The criteria for significant induction of a particular gene were a signal value ≥100 for the objective samples and a fold change ≥ 2^1^. The criteria for significant expression reduction for a particular gene was a signal value ≥100 for the control samples and a fold change ≤2^−1^.

### RNA-sequencing

RNA quality was assessed by Nanodrop measurement (Thermo Fisher Scientific). RNA-sequencing (RNA-Seq) libraries were prepared using TruSeq Rapid PE Cluster Kit and TruSeq Rapid SBS kit (Illumina). The libraries were sequenced on Illumina HiSeq 2500 using a read length of 2 × 150 bp. RNA-seq reads were demultiplexed using CASAVA v1.8.2 and aligned to human transcriptome (UCSC gene) and genome (GRCh37/hg19) references respectively using BWA [51]. After transcripts’ coordinates were converted to genomic positions, an optimal mapping result was selected either from transcript or genome mapping by comparing the minimal edit distance to the reference. Local realignment was performed within in-house short reads aligner with smaller k-mer size (k = 11). Finally, fragments per kilobase of exon per million mapped fragments (fpkm) values were calculated for each UCSC gene while considering strand-specific information. Summary sequencing statistics and data quality metrics are shown in Supplementary Table 17.

### Quantitative real-time PCR (qPCR)

First-strand cDNA was synthesized from total RNA with oligo dT primers using the SuperScript® III First-Strand Synthesis System (Thermo Fisher Scientific). qPCR was performed using SYBR green PCR Master Mix (Perkin-Elmer Life Sciences) in 384-well plates using the CFX384 Real-Time system (Bio-Rad) [49]. All reactions were performed in triplicate. The relative amount of all mRNAs was calculated using the comparative CT method. GAPDH mRNA was used as the invariant control for all studies. Primers used for qPCR are listed in Supplementary Table 18.

### Immunoblotting

Cells on 6-well plates were lysed with 300 μl of RIPA buffer (Thermo Scientific) supplemented with complete protease inhibitor cocktail (Roche). Protein concentration was determined by the Bradford method (Bio-Rad). Whole-cell samples were resolved by SDS-polyacrylamide gel electrophoresis using 10% Ready Gel (Bio-Rad), then electro-transferred to Immobilon transfer membranes (Millipore). Membranes were blocked with 5% skim milk (Wako Pure Chemical Industries, Ltd.) in TBS with 0.1% Tween-20 for 30 min at room temperature. The blot was probed with primary antibodies overnight at 4°C and then incubated with anti-IgG horseradish peroxidase-conjugated antibodies (Sigma-Aldrich) for 1 h at room temperature. Proteins were detected using SuperSignal CL-HRP Substrate System (Thermo Fisher Scientific) according to the instructions of the manufacturer. Immunoreactive protein bands were documented using a Bio-Rad ChemiDoc XRS+ system (Bio-Rad). Antibodies used for immunoblot are listed in Supplementary Table 19.

### Cell growth assay

Cells were seeded in 6-well plates at a concentration of 1 × 10^5^ cells per well. Twenty-four hours later, cells were treated with various siRNAs, and cultured for an additional 2 (HUVECs) or 6 days. Media and reagents were replaced at day 3. At the end of the culture period, the cells were trypsinized and counted with a hemocytometer.

### Cell invasion assay

Cell invasion ability was evaluated using 24-well BD BioCoat Matrigel Invasion Chambers (BD Bioscience) as per the manufacturer guidelines. 2 × 10^5^ cells were added to the upper wells onto an 8 μm pore size PET membrane coated with a thin layer of matrigel basement membrane matrix. After 16 h of incubation, cells were stained with Diff Quick stain (Sysmex) after removing the non-migrated cells from the top of the membrane with Q-tips. After air-drying, the cells that had migrated to the underside of the filter were counted using a light microscope (Leica) in four randomly selected fields (magnification: 40×). Each assay was performed in triplicate.

### Mouse xenograft studies

Five-week-old male SCID mice were obtained from Charles River Laboratories (Japan) and maintained in a laminar air flow cabinet under specific pathogen-free conditions. After one week of adaptation, castration was performed under anesthesia by making a small incision in the scrotum to remove each testicle after ligation of the cord. Mice were allowed to recover for an additional 1 week before inoculation with PCa tumor cells: LNCaP (n = 7 mice), LNCaP-SF shControl (n = 14 mice), LNCaP-SF shNONO-1 (n = 14 mice) or shNONO-2 (n = 14 mice) to generate a xenograft model of human prostate cancer. To this end, a total of 100 μL of cell suspension containing 5.0 × 10^6^ cells and 50% of matrigel was injected subcutaneously into the dorsal flanks of the mice via a 29-gauge needle. Body weight and tumor size were measured every 3 or 4 days. Caliper-measured tumor volume was calculated by the formula: larger diameter × (smaller diameter)^2^ × 0.5 [26]. The mice used in the current study were maintained and sacrificed in accordance with the guidelines of the Committee on Animal Experimentation of the University of Tokyo.

### PSA enzyme immunoassay

Mouse serum was frozen at −80°C until PSA measurement, performed using a human PSA Enzyme Immunoassay kit (Markit M PA, Dainippon Pharmaceutical).

### Statistical analyses

Homogeneity in variance was evaluated by Bartlett's test followed by parametric or non-parametric Dunnett's multiple comparison test. Significance was assessed at ^*^P < 0.05, ^**^P < 0.01.

## SUPPLEMENTARY MATERIALS FIGURES AND TABLES















































## Abbreviations

PCa: prostate cancer
CRPC: castration-resistant prostate cancer
LNCaP-SF: LNCaP steroid free
