# Supplementary material for: Overexpression of p54nrb/NONO induces differential EPHA6 splicing and contributes to castration-resistant prostate cancer growth
Source: Oncotarget. 2018 Jan 8;9(12):10510–24. doi: 10.18632/oncotarget.24063 (PMC5828187; doi:10.18632/oncotarget.24063)
Supplement: Supplementary file 1 [file oncotarget-09-10510-s001.pdf]

Overexpression of p54^{nrb}/NONO induces differential *EPHA6* splicing and contributes to castration-resistant prostate cancer growth

SUPPLEMENTARY MATERIALS

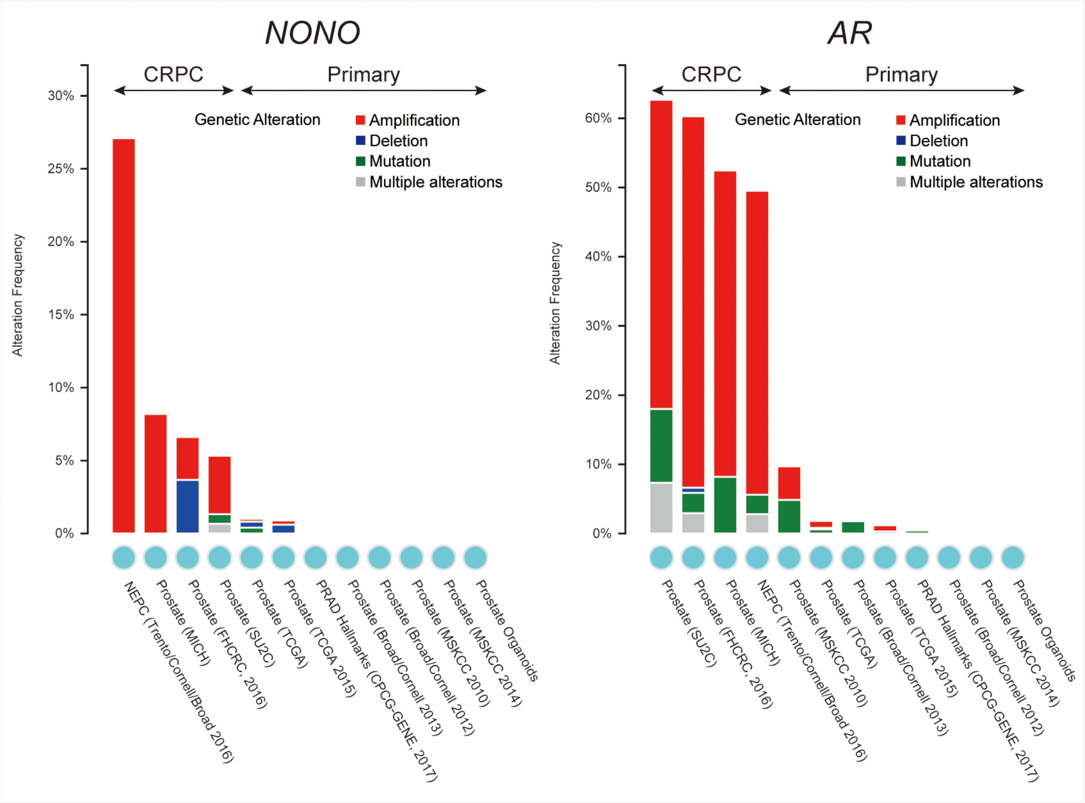

Supplementary Figure 1: p54^{nrb}/NONO gene amplification in CRPC. Summary graph of *NONO* and *AR* alterations (shown in different colors) in individual studies deposited in the cBioPortal.

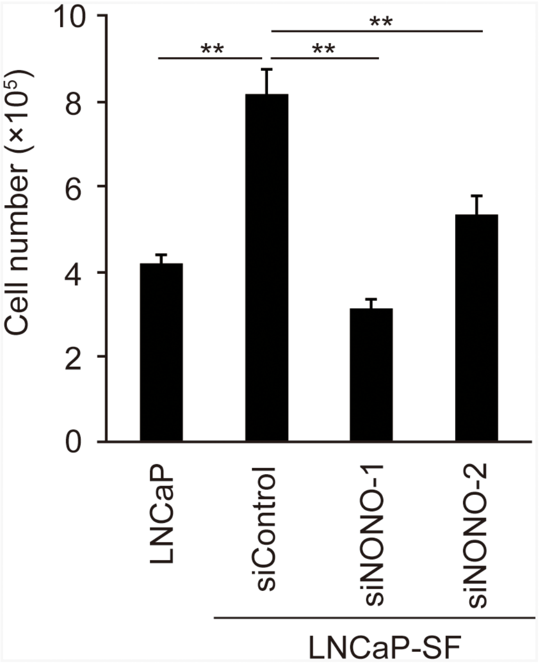

Supplementary Figure 2: p54nrb/NONO silencing reduces LNCaP-SF cells growth. Effect of NONO siRNAs on LNCaP-SF cell growth rate under androgen deprivation condition. SiNONOs significantly reduced cell growth rate of LNCaP-SF cells under androgen deprivation condition.

Supplementary Table 1: Targeting sequences of siRNAs

See Supplementary File 1

Supplementary Table 2: Targeting sequences of shRNAs

See Supplementary File 2

Supplementary Table 3: Summary of exome sequencing

See Supplementary File 3

Supplementary Table 4: Summary of RNA sequencing

See Supplementary File 4

Supplementary Table 5: List of primers used in this study for RT-PCR

See Supplementary File 5

Supplementary Table 6: Antibodies used for western blot analysis

See Supplementary File 6

Supplementary Table 7: Upregulated probe sets in LNCaP-SF and siControl

See Supplementary File 7

Supplementary Table 8: Downregulated probe sets in LNCaP-SF and siControl

See Supplementary File 8

Supplementary Table 9: Upregulated probe sets in siAR

See Supplementary File 9

Supplementary Table 10: Downregulated probe sets in siAR

See Supplementary File 10

Supplementary Table 11: Overapping probe sets between LNCaP-SF differentially expressed and siAR-regulated genes

See Supplementary File 11

Supplementary Table 12: Number of somatic variations

See Supplementary File 12

Supplementary Table 13: Summary of Copy Number Variations (CNV)

See Supplementary File 13

Supplementary Table 14: Probe sets located in Xq11.22-q21.33 region

See Supplementary File 14

Supplementary Table 15: Probe sets located in Xq11.22-q21.33 region (Average difference_LNCaP-SF ≧ 100)

See Supplementary File 15

Supplementary Table 16: Upregulated genes in LNCaP-SF

See Supplementary File 16

Supplementary Table 17: Downregulated genes in LNCaP-SF

See Supplementary File 17

Supplementary Table 18: Upregulated genes in siNONO

See Supplementary File 18

Supplementary Table 19: Downregulated genes in siNONO

See Supplementary File 19

Appendix for Supplementary Table 12: Mutations

See Supplementary File 20

Appendix for Supplementary Table 13.1: Total Copy Number Variations (CNV)

See Supplementary File 21

Appendix for Supplementary Table 13.2: Allelic Copy Number Variations (CNV)

See Supplementary File 22
